# Remission of irreversible aripiprazole-induced tardive dystonia with clozapine: a case report

**DOI:** 10.1186/s12888-015-0644-1

**Published:** 2015-10-19

**Authors:** Soohyun Joe, Jangho Park, Jongseok Lim, Choongman Park, Joonho Ahn

**Affiliations:** Department of Psychiatry, Ulsan University Hospital, University of Ulsan College of Medicine, Ulsan, Republic of Korea; Department of Psychiatry, Ulsan University Hospital, 877 Bangeojinsunhwan-doro, Dong-gu, Ulsan, Republic of Korea

**Keywords:** Aripiprazole, Tardive dystonia, Clozapine

## Abstract

**Background:**

Aripiprazole can cause irreversible tardive dystonia in some individuals, and additional intervention is sometimes needed. Here, we report the first case of aripiprazole-induced irreversible tardive dystonia in which complete recovery of motor function was achieved using the antipsychotic drug clozapine.

**Case presentation:**

A 24-year-old man with bipolar disorder was treated with aripiprazole and gradually developed tardive dystonia. Thorough medical and neurological examinations were performed to rule out other possible causes of tardive dystonia. Clozapine was administered when the patient did not improve following long-term withdrawal of aripiprazole or adjuvant medications. Before administration of clozapine, the patient was experiencing severe dystonia as assessed by the Extrapyramidal Symptom Rating Scale. Dystonic symptoms began to improve about 1 month after starting administration of clozapine and were completely resolved 3 months after clozapine administration.

**Conclusions:**

Clinicians should note the risk of aripiprazole-induced tardive dystonia and consider clozapine as an alternative and effective treatment modality in cases of irreversible tardive dystonia, particularly when concomitant treatment of psychotic symptoms is required.

## Background

Tardive dystonia is a serious movement disorder that occurs in patients treated with antipsychotics [1], and has been shown to develop in about 3 % of patients who have had long-term exposure to antipsychotics [2]. If untreated, this condition can often cause considerable distress and can lead to permanent debilitation [3, 4]. Chronic dopamine receptor antagonism is known to be strongly associated with the manifestation of tardive dystonia symptoms [5]. The low risk of tardive dystonia for atypical antipsychotics is thought to result from their weak affinity for dopamine receptors [6, 7].

Aripiprazole is an atypical antipsychotic with unique pharmacological properties. Because of its partial antagonistic activity on dopamine D2 receptors, aripiprazole is associated with an especially low risk of developing tardive dystonia. Moreover, some reports have described the use of aripiprazole in the treatment of tardive dystonia [8, 9]. Nonetheless, aripiprazole has also been shown to cause tardive dystonia in some cases, and motor symptoms of aripiprazole-induced tardive dystonia can sometimes be reversed by withdrawal of aripiprazole and addition of anticholinergic medications [10–13]. However, in some cases, neither withdrawal of medication nor treatment with drugs, such as trihexyphenidyl, have been shown to be effective in alleviating symptoms [10, 14, 15].

Some studies have reported improvement in symptoms of tardive dystonia by switching from a typical antipsychotic to an atypical antipsychotic [16], such as clozapine. However, attempts to assess the efficacy of clozapine on motor symptoms in patients with tardive dystonia are conflicting and inconclusive. Many previous studies examining the effects of switching to clozapine for antipsychotic-induced tardive dystonia have reported a reversal of motor symptoms [17, 18]. However, it is not clear whether discontinuation of the original antipsychotic treatment or the switch to clozapine leads to improvements in movement symptoms.

Here, we present the first case of an aripiprazole-treated patient who developed tardive dystonia and experienced reversal of symptoms with clozapine treatment. The patient gave written informed consent for publication.

## Case presentation

Mr. A, a 24-year-old man with bipolar disorder, presented with irritable mania in July 2009. His father had schizophrenia, and his sister had a schizoaffective disorder. He had never taken antipsychotics prior to this manic episode. Aripiprazole (5 mg/d) was administered to manage the manic symptoms, and gradual titration was tried. However, the patient and his family refused to take more than 5-mg aripiprazole because of a concern of adverse events by dose escalation. His manic symptoms slightly improved by December 2009. Though he was still manic, he was not aggressive enough to be suicidal or violent. Between September and October 2010, an agreement was obtained from the patient and his family for dose escalation, and gradual titration up to 15 mg/d was achieved by October 2010. The patient’s manic symptoms were partially remitted by December 2010, and sodium valproate was later added to the treatment strategy in August 2011 (titrated to 800 mg/d by November 2011) to improve residual manic symptoms. Propranolol (20 mg/d) was also added to the treatment regimen because the patient complained of a twitching sensation in the periocular area (September 2011). Propranolol was titrated up to 40 mg/d by December 2011, and the patient reported improvement in his twitching symptom (January 2012).

In July 2012, 3 years after the initiation of drug treatment, the patient began complaining of neck stiffness. By this time, he was taking daily doses of aripiprazole (15 mg), sodium valproate (800 mg), and propranolol (30 mg). He was not taking any other medication that could potentially cause tardive dystonia, including herbal medication, within 1 month before dystonia developed. Benztropine (4 mg) and valium (2 mg) were prescribed to treat his neck stiffness, but these were not effective. By September 2012, he had gradually developed cervical dystonia with right-side convexity and neck hyperextension. The patient could voluntarily reposition himself at first. However, 1 month later, this had become impossible. His symptoms satisfied the following diagnostic criteria for tardive dystonia [19]: (1) he had dystonia; (2) he developed dystonia during antipsychotic treatment; (3) Wilson’s disease was ruled out, and there were no other neurological signs to suggest one of the many causes of secondary dystonia; (4) there was no family history for dystonia. He showed slight abnormalities in liver function (Aspartate transaminase 27 IU/L, Alanine transaminase 52 IU/L), ceruloplasmin (15 mg/dL), and 24-h urine copper tests (77.99 μg/day); however, further studies, including ultrasonography of the liver and ophthalmologic examination did not reveal or confirm Wilson’s disease. Otherwise, there were no abnormal findings on hematology tests, biochemistry tests, or serum valproate levels. A thorough neurologic examination confirmed that he had no other movement disorders. No abnormalities were detected in brain magnetic resonance imaging (MRI).

Aripiprazole was continued for about 6 months even after the onset of dystonia, because he and his family refused to change medication because of fear of adverse events by new medication. Because his dystonia became worse as time progressed we decided to discontinue aripiprazole. The daily dose of aripiprazole was tapered and discontinued over 11 weeks after the appearance of torticollis. By January 2013, administration of aripiprazole ceased completely. The patient reported experiencing an irritable mood and frequent anger outbursts in February 2013. No other accompanying tardive syndromes other than neck tardive dystonia were observed in this patient. To treat tardive dystonia and to prevent relapse of mania, clozapine treatment was recommended to the patient in March 2013; however, he refused to take clozapine because of fear of developing agranulocytosis. By April 2013 the patient was experiencing severe dystonia (Score of Dystonia = 10) based on the Extrapyramidal Symptom Rating Scale (ESRS). Despite withdrawal from aripiprazole, the dystonic symptoms persisted, and the index of severity, measured by the ESRS, was unchanged, even 8 months after complete discontinuation of aripiprazole. His neck dystonia caused severe distress and impaired social and occupational function. He was concerned about what people would think about his posture, and tended to stay home from work and social events. He ultimately quit his position at his university. Moreover, the patient continued to experience fluctuating irritability and uncontrolled anger.

We decided to administer clozapine for the treatment of tardive dystonia because it was a promising candidate for improving motor symptoms [14, 20] and was expected to control his manic symptoms. The potential efficacy of clozapine in treating aripiprazole-induced tardive dystonia had not yet been previously reported. The starting dose of clozapine was 12.5 mg/day, and the dose was gradually titrated weekly up to 87.5 mg/day until August 2013. Dystonic symptoms began to improve 1 month after clozapine was started, and tardive dystonia was completely resolved 3 months after clozapine administration was started (September 2013). His ESRS score reached zero. At that time, the dose of clozapine was 87.5 mg/day. Additionally, by November 2013 his manic symptoms dramatically improved, and his daily dose of clozapine was 100 mg/d.

## Conclusions

In the present case, the occurrence of tardive dystonia in an antipsychotic-naïve patient strongly suggested that aripiprazole was the causal agent. While he showed tardive dystonia, his father and sister, who are on other antipsychotics, did not experience tardive dystonia, and thus, there is no history of familial dystonia. To our knowledge, concomitant medications, i.e., sodium valproate and propranolol, have not been reported to cause tardive dystonia, and in fact, valproate was reported to improve tardive syndrome in an open trial [20]. Neurologic examinations did not show other causes of dystonia. This patient has several well-known risk factors, e.g., young, male, and was diagnosed with affective disorder [1]. Several hypotheses, such as interactions between antipsychotics and antidepressants, or short-term use of high dose antipsychotics in affective disorder have been suggested for high risk of tardive dystonia in affective disorder [21]. Among them, vulnerability of the brain due to cyclic mono- and catecholamine activity during mood swings may account for tardive dystonia in this case.

This patient was a good candidate for clozapine therapy because he needed medication to concurrently improve tardive dystonia and to alleviate his manic symptoms. Other therapies for tardive dystonia are not known to be effective in manic symptoms. In addition, he is young and duration of tardive dystonia was relatively short (about 8 months). In a recent review, clozapine was reported to be more beneficial in treating tardive dystonia in patients who are young and have shorter duration [22]. In this case, tardive dystonia persisted and was unchanged for a relatively long period, despite withdrawal of aripiprazole, and the symptoms were rapidly and completely reversed after clozapine administration. This indicates that clozapine reversed aripiprazole-induced tardive dystonia in this case.

Previous case studies [10, 14] reported that antipsychotic-naïve patients developed aripiprazole-induced tardive dystonia (Table 1). Among them, spontaneous reversal of tardive dystonia symptoms by antipsychotic withdrawal and the passage of time occurred in only one case [10]. In another case, dystonia persisted for over 18 months, and medications, including trihexyphenidyl and botulinum toxins, were not effective in alleviating symptoms. Deep brain stimulation therapy finally produced an improvement in this patient [10]. In another case, dystonia was reported to have persisted over a 2-month observation period [14].

**Table 1:** Case studies of aripiprazole induced-tardive dystonia

	Oommen et a.l. [12]	Lungu et al. [10]	Pinninti et al. [13]	Matsuda et al. [11]	Lungu et al. [10]	Sato et al. [15]	Friedman [14]
Sex	Female	Female	Male	Female	Male	Male	Female
Age	25	19	35	23	62	19	53
Diagnosis	Schizoaffective disorder	Anxiety disorder & violent outburst	Schizoaffective disorder	Schizophrenia	Post- traumatic stress disorder	Schizophrenia	Depression
Involved body part	Trunk (lumbar)	Head & trunk	Trunk (thoracic)	Larynx	Periocular area, neck	Wrist, elbow, shoulder	Trunk, pelvis
Reversibility	(+)	(+)	(+)	(+)	(−)	(−)	(−)
Effective treatment	Trihexyphenidyl	(−)	(−)	Quetiapine	Deep Brain Stimuation^a^		Tetrabenazine^b^
Duration of aripiprazole medication	2 months	3 years	About 8 weeks	7 months	18 months	8 months	2 years
Previous antipsychotics use	(+)	Drug naïve	(+)	(+)	Drug naïve	(+)	Drug naïve
Examinations	(−)	(−)	(−)	Lab, brain MRI^c^	(−)	Lab, brain MRI^c^	(−)

^a^Unresponsive to trihexyphenidyl, clonazepam, baclofen, and botulinum

^b^Partially effective

^c^Results: normal

Withdrawal of aripiprazole alone was not enough to reverse tardive dystonia in previous cases [10, 11, 14]. Trihexyphenidyl and quetiapine were used in those cases. However, it is not clear whether the medication or withdrawal of aripiprazole reversed the motor symptoms. Unlike previous reports, the present case study had a relatively long interval between withdrawal of aripiprazole and the onset of clozapine administration, supporting the idea that clozapine exerted therapeutic effects in aripiprazole-induced tardive dystonia. Although the use of clozapine has some limitations because of the risk of agranulocytosis, it can be an effective alternative treatment in the management of irreversible tardive dystonia.

The exact pathophysiology of tardive dystonia remains uncertain. Tardive dystonia has been ascribed to supersensitivity of dopamine receptors, particularly D2, after long-term blockade [22]. However, this may not be the only explanation for the development of tardive dystonia. Imbalance between direct and indirect pathways of the basal ganglia was recently mentioned as an underlying mechanism for tardive dystonia [23]. In addition, maladaptive synaptic plasticity in corticostriatal transmission and oxidative stress, both provoked by chronic D2 blockade, were proposed to be responsible for tardive dystonia [24, 25]. Also 5-HT_2A/2C_ receptors are probably involved in the pathogenesis of tardive dystonia [26]. 5-HT_2A_ and 5-HT_2c_ antagonism enhance dopamine release, and the low risk of tardive dystonia by clozapine and quetiapine is attributed to these properties.

The relatively low risk of tardive dystonia with aripiprazole is attributed to the unique partial agonistic activity on D2 receptor [18]. However, aripiprazole has one of the highest D2 receptor affinities at a therapeutic dose [18]. Theoretically, long-term exposure of aripiprazole to D2 receptors may cause irreversible hypersensitivity of D2 receptors. A model explaining the development of the tardive syndrome suggests that chronic D2 (indirect inhibitory pathway) receptor occupancy by long term antipsychotic medication may sensitize D1 receptors and may lead to an imbalance of D1 and D2 striatal outputs. According to this model, a D1 antagonist may be an effective treatment for tardive syndrome [27]. Clozapine has relatively high D1 and low D2 receptor occupancy. [28]. This may explain the efficacy of clozapine for tardive dystonia in our case. Also, aripiprazole has partial antagonistic properties at 5-HT2A/2C receptors, which is very unique and another difference of aripiprazole and clozapine. This may be involved in reversal of aripiprazole induced tardive dystonia by clozapine in this case. [29].

To date, there is no established treatment to cure this severe form of movement disorder [22]. This case report suggests that clozapine may be effective in treating irreversible aripiprazole-induced tardive dystonia. Because this is a case report, overgeneralization of the results should be avoided, and more systematic research, such as randomized, controlled, blinded studies, should be carried out. As a relatively new antipsychotic, the therapeutic indications of aripiprazole are broadening, and the occurrence of tardive dystonia among patients taking aripiprazole may increase. Clinicians should be aware of the risk of aripiprazole-induced tardive dystonia and should consider clozapine treatment in irreversible cases, especially when concomitant treatment for psychotic symptoms is needed.

### Consent

The patient has given his written informed consent for this case report to be published. Before he gave his written informed consent for this case report to be published, he was in healthy enough mental and physical condition that would have allowed them to provide informed consent for publication with his free will, good enough intelligence for decision making ability and clear consciousness.

## Abbreviations

MRI: Magnetic Resonance Imaging
ESRS: Extrapyramidal Symptom Rating Scale
